# Household Hints and Recipes

**Published:** 1878-04

**Authors:** 


### Household Hints & Recipes.

RED INK.—The following recipe for a beautiful red Ink is given by Metra, of Paris: Dissolve 25 parts, by weight, of saffranine in 500 parts warm glycerine, then stir in carefully 500 parts alcohol and an equal quantity of acetic acid. It is then diluted with 9,000 parts water, in which is dissolved a little gum arabic.

---

PORCELAIN CEMENT.—Transparent gelatine, or good glue, is cut into small pieces, covered with a little acetic acid, and gently heated until it has melted together to a clear homogeneous mass, which hardens on cooling. When using it the warmed broken surfaces are brushed over with a small quantity of the cement, previously liquified by warming, firmly pressed together, and allowed to remain at rest for about twenty-four hours.

---

TO SILVER IRON.—To silver cast iron, 15 grains of nitrate of silver are dissolved in 250 grains of water, and 30 grains cyanide of potassium are added; when the solution is complete, the liquid is poured into 700 grains of water, wherein 15 grains of common salt have been previously dissolved. The cast iron intended to be silvered by this solution should, after having been well cleaned, be placed for a few minutes in a bath of nitric acid of 1.2 specific gravity just before being placed in the silvering fluid.

OIL OF EGGS.—Extraordinary stories are told of the healing properties of a new oil which is easily made from the yolk of hen's eggs. The eggs are first boiled hard, and the yolks are then removed, crushed, and placed over a fire, where they are carefully stirred until the whole substance is just on the point of catching fire, when the oil separates, and may be poured off. One yolk will yield two teaspoonfuls of oil. It is in general use among the colonists of South Russia as a means of curing cuts and bruises, etc.

---

CEMENT FOR MENDING RUBBER.—The best and simplest is probably a solution of virgin caoutchouc in bisulphide of carbon. The varnish, if any, should first be removed from the surface near the place to be mended. Then a piece of cloth of suitable shape and color, is smeared on both sides with the cement, and applied with gentle pressure while the sides of the rent are kept close together. The rubber solution has the consistence of a stiff jelly, and owing to the volatility of the menstruum, dries in a very short time.

---

IMPROVED BLACK WRITING INK.—This ink is prepared by digesting for 24 hours, at a gentle heat, 7½ ounces of powdered nut-galls in a pint of alcohol of 82° per cent., and in another vessel, 2¼ ounces of sulphate of iron, and the same quantity of gum arabic, with 3 pints of pure water. These two liquids, passed through a flannel strainer, are then to be mixed and allowed to settle for eight days, and again strained. The ink thus obtained does not form any deposit, nor does it mould, and is more permanent than any other.

---

MINCE PIES.—Boil four or five pounds top sirloin of beef; keep the soup for the pies. After chopping the meat fine, chop as much of tart apples; let it measure as much as the meat when chopped; add two pounds of stoned raisins, two pounds of currants washed and dried, one-half pound citron cut fine, all the soup, more than a quart, one tablespoonful cloves, one ounce grated nutmegs, a pint of black molasses, one quart cider, one and one-half pints brandy, one pound suet. After mixing and stirring well try it, and add such ingredients as are needed.—*Mrs. T.*

---

KEROSENE LINIMENT.—Dr. A. C. Hobbs, in the *Louisville Medical News*, recommends the following:

| | | |
|---|---|---|
| Kerosene oil................ | 2 | ounces. |
| Tincture of opium............. | 4 | drachms. |
| " " arnica............ | 5 | " |
| " " stramonium........ | 4 | " |
| Aromatic spirit of ammonia.... | 6 | " |
| Spirit of Camphor............ | 5 | " |
| Oil of origanum.............. | 4 | " |
| Chloroform.................. | 1 | ounce. |

Mix. An excellent liniment for sprains, bruises, soreness, and nervous pains; it is good for man and beast.

---

COFFEE AND MILK AS A REMEDY FOR CANINE DISTEMPER.—It will be interesting to lovers of the canine species to hear of a simple remedy for distemper. At the quarterly meeting of the Scottish Metropolitan Veterinary Medical Society, Mr. Baird mentioned the case of a colly dog in the last stage of the disease, and which its owner had determined to destroy. Shortly after being treated with doses of strong coffee and a little sweet milk, the animal, however, so far recovered as to be able to stand and walk. The chairman of the meeting said the case seemed almost unique.—*London Lancet.*

---

A NEW LIQUID GLUE.—To produce good glue size, dissolve in a copper pan, heated by indirect steam, 4½ to 5 lbs. of soda in 20 lbs. to 24 lbs. of boiling water; then add to it, stirring well at the time, 30 lbs. of powdered resin, keeping the whole continually boiling until all the resin is perfectly dissolved. This soda-resin composition, dissolved in the proportion of 1 lb. of resin to 30 lbs. to 40 lbs. of water, is to be mixed well together with a glue solution, made by dissolving 10 lbs. of glue in about 30 lbs. to 40 lbs. of water; then boil up both solutions together for about 10 minutes, after which run it through a fine sieve or filter, and it is then ready for use. The best proportions for mixing the vegetable and animal sizes are, for 1½ parts resin add 11 parts glue.

---

ARTIFICIAL MILK.—Some patients cannot take milk, and yet need to be fed on a liquid food. The following substitute will meet such cases. Pour a pint of rice water upon a tablespoonful of a mixture of fine almond flour (4 oz.) and prepared sugar (2 oz.) The latter consists of sugar of milk 13 oz., powdered lump sugar 2 oz., and 1 oz. of a combination of which this is the formula: Chloride of potassium, 6 oz.; phosphate of soda, 3 oz.; phosphate of magnesia and phosphate of iron, 1 dr. The rice water should be poured on boiling hot and the mixture well stirred, after this it should cool, and then be strained through a sieve.

---

VERMIN ON POULTRY.—John E. Roberts, of this city, in the *Southern Poultry Journal*, says:

"Many fanciers use the carbolic (or carbolated) powder in order to rid their fowls of lice and mites. It is considered the very best of remedies. My plan is one which, I think, is used by no other breeder; has never failed me in completely ridding my fowls of every insect, and has demonstrated to me its infallibility. It is simply the use of oil of sassafras mixed with sweet oil. To one ounce of oil of sassafras put five or six of sweet oil, and apply a small quantity to different parts of the body of the fowl, selecting those points where the vermin would be most apt to hide.

"In applying, the preparation, I fill with it a small oil-can, so that I can force out as much or little of the oil as I wish. A very small bit can be made to go a great ways, for one drop can be rubbed over two or three inches of space, and is no more trouble to apply than the various insect powders. I use sweet oil, because of its curative powers, but any kind of grease, no matter what, will do to mix with the oil of sassafras. The oil of sassafras is the eradicator, the oil merely the vehicle. I believe common sassafras tea would be wonderfully efficacious.

"Make it in a large pot, then, after allowing it to cool, dip the fowls in bodily. In one second the lice will be dead, and in ten seconds the fowl will be perfectly dry, if placed in the sunshine. It is hard to form an idea of the magical effect produced by the oil of sassafras. I have never tried the remedy in greater attenuation than that mentioned (one part to five or six), but believe that it would be equally good if composed of one ounce of oil of sassafras to ten or twelve of any other oil or grease."—*Rural New Yorker.*

---

REMOVAL OF STRONG ODORS FROM THE HANDS.—Ground mustard, mixed with a little water, is an excellent agent for cleansing the hands after handling odorous substances, such as cod-liver-oil, musk, valerianic acid, and its salts. Scale pans and vessels may also be readily freed from all odor by the same method.

A. Huber states that all oily seeds, when powdered, will answer this purpose. The explanation of this action is somewhat doubtful, but it is not improbable that the odorous bodies are dissolved by the fatty oil of the seed, and emulsionized by the contact with water. In the case of bitter almonds and mustard, the development of ethereal oil, under the influence of water may, perhaps, be an additional help to destroy foreign odors. The author mentions that the smell of carbolic acid may be removed by rubbing the hands with damp flax-seed meal, and that cod-liver-oil bottles may be cleansed with a little of the same or olive oil.

---

**To Crystallize Grasses and Flowers.**

Alum, 18 ozs.
Soft water, 2 pints.

Dissolve the salt by means of a gentle heat, and allow the solution to cool down. When the liquor is almost cold, but before it begins to crystallize, suspend in it the objects to be covered with crystals by means of a small thread or twine attached to a small stick laid horizontally across the aperture of the vessel. After twenty-four hours the objects are withdrawn from the solution, and dried in ihe shade.

